# Optimizing tropical dairy goat diets: balancing rumen degradable protein, non-fiber carbohydrates, and sulfur requirements

**DOI:** 10.5713/ab.24.0155

**Published:** 2024-10-24

**Authors:** Idat Galih Permana, Annisa Rosmalia, Febby Yustika Anggarini, Despal Despal, Toto Toharmat, Dwierra Evvyernie

**Affiliations:** 1 Department of Animal Nutrition and Feed Technology, Faculty of Animal Science, IPB University, Bogor 16680, Indonesia

**Keywords:** Cassava, Dairy Goat, Feed Efficiency, Heat Treatment, Non-Fiber Carbohydrate, Rumen Degradable Protein

## Abstract

**Objective:**

This study aimed to investigate the effects of rations incorporating rumen degradable protein (RDP), non-fiber carbohydrate (NFC), and sulfur on nutrient utilization, milk production, milk quality, and the economic aspects of dairy goats.

**Methods:**

In the first study, five treatments were tested in a block-randomized design to examine *in vitro* fermentability and digestibility. Treatments included P0 (control diet), P1 (P0+7.5% cassava-NFC), P2 (P0+7.5% cassava-NFC and 5% soybean), P3 (P0+7.5% cassava-NFC and 5% autoclaved soybean), and P4 (P0+7.5% cassava-NFC, 5% autoclaved soybean, and 0.1% sulfur). In the second study, sixteen lactating Saanen-Ettawa crossbreed dairy goats (initial milk production = 0.97±0.25 L/head/d, 30 DIM; body weight = 44.44±7.20 kg) were assigned into four groups and fed treatment diets: R0 (basal diet), R1 (R0+12% autoclaved soybean), R2 (R0+12% autoclaved soybean and 9% cassava-NFC), and R3 (R0+12% autoclaved soybean, 9% cassava-NFC, and 0.11% sulfur). The diets were offered for 7 weeks with a two-week adaptation period. Parameters observed include milk production and quality, milk fatty acids, blood hematology and metabolites, and economic aspects. The study used a block randomized design with initial weight as a block.

**Results:**

The treatment diets in the first study had no effect on *in vitro* fermentability and digestibility. Treatments R2 and R3 resulted in higher milk production than R0 and R1. Milk quality remained consistent across treatments, while solid non-fat, lactose, and protein was higher in R2 and R3. Blood hematology was unaffected by the treatments. Nutrient efficiency and income over feed cost were enhanced by R2 and R3 treatments.

**Conclusion:**

Protected RDP using autoclaved soybean and cassava-NFC maintained *in vitro* digestibility, even though it did not improve *in vitro* fermentability. Precision dairy ration based on RDP, NFC, and sulfur positively impacts milk production, nutrient efficiency, and animal health in dairy goats.

## INTRODUCTION

The dairy goat sector plays a crucial role globally in meeting the demand for high-quality milk and dairy products, particularly in tropical regions with challenging environmental conditions. Achieving and maintaining high health and productivity levels in dairy goats requires a deep understanding of their nutritional needs. In Indonesia and other Southeast Asian countries, the dairy goat population is growing rapidly to meet increasing demand. Smallholder farms typically manage these dairy goat systems, relying on a combination of forages and concentrates provided by local cooperatives. However, this diet often falls short of meeting the nutritional requirements of lactating goats, leading farmers to supplement with high-value agro-industrial byproducts like tofu waste, which significantly increasing the concentrate portion [1]. This heavy reliance on concentrates raises concerns about cost-effectiveness, animal health, and overall farm efficiency. Adhering to sustainable livestock practices involves precisely meeting livestock nutritional needs to improve feed efficiency, leading to economic gains while minimizing environmental impact.

In Indonesia, the existing dairy livestock ration formulation only considers crude protein (CP), total digestible nutrients (TDN), and Ca and P minerals. However, achieving precise nutrient requirements poses challenges as dairy livestock require nutrients for host animals and rumen microbial development. Rumen microbes require fermentation products, such as ammonia (NH_3_) and volatile fatty acids (VFA), to be synchronized in their release for efficient synthesis of microbial protein (MP). NH_3_ in the rumen primarily originates from the breakdown of dietary protein and urea by rumen microbes, while VFAs are produced through the fermentation of carbohydrates by rumen microbes, particularly cellulolytic bacteria and protozoa. Easily fermentable carbohydrate components within the rumen can be supplied by non-fiber carbohydrates (NFC) in feed. Rumen degradable protein (RDP) and NFC synchronization in the dairy ration is crucial to optimize MP production, improve rumen microbial activity, and subsequently enhance animal performance. Synchronizing RDP (60%) with NFC (35%) and TDN (68% to 75%) resulted in optimal fermentation products, MP synthesis, and digestibility [2].

Soybean is extensively used as a protein source in ruminant diets, but its protein content undergoes significant degradation in the rumen (RDP = 64.80%), thereby reducing its quality and necessitating protective measures. Previous research has shown that autoclaving effectively improves protein protection [3]. Cassava meal serves as another widely used and economical energy source for ruminants. Our previous study has shown that cassava meal offers a superior NFC source (NFC = 76.71%) compared to corn-NFC (NFC = 70.89%) [4]. This superiority is attributed to cassava-NFC’s rapid degradation in the rumen due to its low amylose-to-amylopectin ratio.

In the context of MP production, it is imperative to account for the sulfur mineral requirements, alongside Ca and P minerals, given their vital role in the synthesis of sulfur-containing amino acids [5]. Sulfur deficiencies are commonly observed in rations. Supplementing 0.2% sulfur mineral in RDP-based rations reduced the protozoa population and increased *in vitro* digestibility. Due to the direct and indirect interactions between protozoa and methanogens, a decrease in protozoa population may correlate with lower methane (CH4) gas emissions [6]. A previous *in vitro* study reported that the incorporation of cassava as an NFC source in RDP-based ration along with sulfur supplementation demonstrated comparable effects to using corn as the NFC source on MP synthesis efficiency, VFA production, and ration digestibility [7]. However, an *in vivo* study on dairy livestock in tropical regions regarding more precise ration reformulation considering RDP, NFC, and sulfur minerals still needs to be improved. Therefore, our objectives were to study the effects of 1) protected-RDP (autoclaved soybean), cassava-NFC, and sulfur-based ration on *in vitro* fermentability and digestibility (exp. 1) protected-RDP (autoclaved soybean), cassava-NFC, and sulfur-based ration on nutrient intake, milk production, and quality, fatty acid composition, blood hematology and metabolites, and economic aspects in dairy goats (exp. 2).

## MATERIALS AND METHODS

This study was reviewed and approved by the IPB University Animal Ethics Committee guidelines (No. 047/KEH/SKE/2021) and followed the animal use procedures in accordance with the Guide for the Care and Use of Laboratory Animals.

### Experiment 1

#### Experimental diet

This study used a randomized block design with five treatment diets, each treatment replicated four times as a block based on different sources of rumen inoculum. The diet in this study was formulated based on the National Research Council (NRC) requirement for dairy cattle [8] and considering the RDP to rumen undegradable protein (RUP) ratio of 60:40 and 30% NFC. The forage-to-concentrate ratio is 50:50. Autoclaved soybean was employed to protect RDP and achieve an optimal RDP and RUP balance ratio in the P1, P2, P3, and P4 treatment diets. The autoclaved soybean was prepared by autoclaving at a temperature of 120°C for 60 min [3]. The treatments consisted of P0 (control diet, without cassava-NFC and autoclaved soybean as protected RDP source), P1 (P0+7.5% cassava-NFC), P2 (P0+7.5% cassava-NFC and 5% soybean), P3 (P0+7.5% cassava-NFC and 5% autoclaved soybean), and P4 (P0+7.5% cassava-NFC, 5% autoclaved soybean, and 0.1% sulfur). The formula and nutrient content of the experimental diet are shown in Table 1.

#### In vitro procedures

The *in vitro* procedure, Tilley and Terry’s method [9], was performed to determine the fermentability and digestibility of the experimental diet. Rumen fluid was collected before morning feeding from a rumen-cannulated Frisian-Holstein bull (524 kg of body weight [BW]) at the Dairy Nutrition Field Laboratory, IPB University. The bull was fed a diet of 2% of its BW, a 60:40 forage to concentrate ratio (dry matter [DM] basis). Subsequently, a mixture comprising 0.5 g of sample, 40 mL of McDougall’s buffer solution, and 10 mL of fresh rumen fluid was introduced into the fermenter tube. Following this, the tube was purged with CO_2_ gas for 10 sec using a syringe to establish an anaerobic environment. The fermenter tube was tightly closed and incubated at 39°C in a shaker water bath for 4 h to measure fermentability and 48 h to estimate digestibility. After 4 h incubation, 1 mL of samples was taken for protozoa analysis. Protozoa were quantified using the Ogimoto and Imai method [10]. Protozoa were enumerated by adding 1 mL of rumen liquor to 1 mL of trypan blue formal saline solution. This mixture was then placed onto a microscope slide and covered with a cover slip. Protozoa were counted using a binocular microscope (XSZ-107BN; Nanjing BW Optics and Instrument Co., Ltd, Nanjing, China) set to 40x magnification.

Rumen pH was assessed using a pH meter electrode (HI98191; Hanna Instruments, Nuşfalău, Romania). The fermentative digestion process was stopped by giving two drops of HgCl_2_ solution. The resulting supernatant was obtained via centrifugation (SL 8R Centifuge; Thermo Fisher Scientific Inc., Bremen, Germany) at 1972×g for 15 min and subsequently stored in a freezer for the subsequent measurement of NH_3_ concentrations. The NH_3_ analysis was conducted using the Conway micro diffusion method [11]. In this method, 1 mL of saturated Na_2_CO_3_ solution and 1 mL of rumen fluid were placed on opposite sides of a Conway dish, with 1 mL of boric acid indicator in the center. The dish was sealed, shaken to mix, and incubated at room temperature for 24 h, after which the absorbed NH_3_ was titrated with 0.005 N H_2_SO_4_ until a red color appeared.

For the digestibility analysis, two drops of HgCl_2_ were added to the tube after 48 h of fermentative digestion. Subsequently, the samples were centrifugated at 1972×g for 15 min to isolate the residue. To this residue, a pepsin-HCl solution (50 mL) was added, followed by incubation at 39°C in a shaker water bath for 48 h. The resulting sample was then filtered through filter paper (Whatman No. 41; Whatman, Maidstone, UK) with the assistance of a vacuum pump. DM and organic matter (OM) were determined by placing the residue in a 105°C oven for 24 h, followed by incineration in a 550°C furnace for four h. The digestibility of dry matter (DMD) and organic matter (OMD) was calculated by subtracting the DM and OM values of the residue from those of the initial samples.

### Experiment 2

#### Animal and experimental diet

The study was conducted at a dairy goat smallholder farm (Harokah Barokah Farm) in Cijeruk, Bogor, West Java, Indonesia (587 masl). This study had a randomized complete block design with 4 treatment diets and 4 blocks. Sixteen multiparous lactating Saanen-Ettawa Crossbreed (Sapera) dairy goats were grouped in 4 blocks based on their initial milk production (0.97±0.25 L/head/d) at thirty days in milk and BW (44.44±7.20 kg). The experimental diet was formulated according to NRC small ruminant [12] guidelines, maintaining a forage-to-concentrate ratio of 40:60, with RDP level set at 60% RDP and NFC level at 30%. Except for the R3 diet, sulfur was added at 0.11% in the form of Na_2_SO_4_. The experimental diet consisted of R0 = basal diet (farm diet); R1 = R0+12% autoclaved soybean; R2 = R0+12% autoclaved soybean and 9% cassava-NFC; and R3 = R0+12% autoclaved soybean, 9% cassava-NFC, and 0.11% sulfur. The diets were offered 3.5% of BW and fed thrice daily at 08.00 am, 01.00 pm, and 04.00 pm. The feed ingredients and nutrient content are shown in Table 2. Animals were kept inside individual cages with separate feeding spaces and had *ad libitum* access to water. The experiment comprised a 2-week diet adaptation period, followed by a 5-week experimental period, with data collection conducted every day during the final week of the experimental period.

#### Environmental climate conditions

The ambient temperature and relative humidity (RH) were recorded daily at 06.00 am, 01.00 pm, and 04.00 pm, employing a digital Thermo-Hygrometer (HTC-1; OneMed, Surabaya, Indonesia). This dataset was utilized to compute the temperature-humidity index (THI) value, following the formula outlined by Mader et al [13].


$$\text{THI}=(0.8\times \text{temperature})+\left[\left(\frac{\text{RH}}{100}\right)\times (\text{temperature}\times 14.4)\right]+46.4$$

#### Sample collection and milk analysis

Feed intake and refusal were measured and sampled for each goat every day during the collection. The samples of both feed and refusal were then subjected to drying at 60°C for 48 h, followed by analysis for DM, OM, CP, ether extract (EE), crude fiber (CF), nitrogen-free extract (NFE), and TDN using the AOAC method [14]. Nutrient intake was subsequently determined based on feed intake and the nutrient composition of the feed.

The goats were subjected to twice-daily milking routines in the morning (07.00 am) and afternoon (03.00 pm), and milk yields were recorded for the entire period. Separate collections of morning and afternoon milk samples (50 mL each) were conducted and subsequently subjected to independent analyses for milk quality, fatty acid profiles, and urea content. Milk quality was measured using Lactoscan SLP: ultrasonic milk analyzer (Milkotronic Ltd., Nova Zagora, Bulgaria). Milk fatty acid profiles were determined through a process involving separation, methylation, and identification using gas chromatography [15]. Milk urea was analyzed through two stages: the preparation stage referred to as the Broderick and Reynal [16] method and the colorimetric method using a spectrophotometer. For milk urea measurement, 5 mL of milk samples were put into a tube, and then 5 mL of 25% trichloroacetic acid solution (weight per volume) was added. Next, the sample was homogenized using a vortex for one minute and allowed to stand at room temperature (25°C) for 30 min. The sample was filtered using filter paper Whatman No. 1. The filtrate was analyzed using a diagnostic kit (urea liquicolor; Human, Wiesbaden, Germany) and subsequently measured using a spectrophotometer (Genesys 10S UV-VIS; Thermo Scientific, Waltham, MA, USA) at a wavelength of 578 nm. The health index of milk fatty acids was determined using the indicators the atherogenicity index (AI), the hypocholesterolemic/hypercholesterolemic (HH), and the Δ^9^-desaturation index (DI) [15].

#### Hematology and blood metabolites analysis

At the end of the experiment, blood samples (3 mL) were obtained prior to morning feeding through jugular vein puncture. The blood samples were promptly deposited into an anticoagulant tube (ethylenediaminetetraacetic acid) and stored in a cooled icebox. Blood samples were separated for hematology and metabolite analysis. Hematological, including red blood cells (RBC), white blood cells (WBC), hemoglobin, and hematocrit. Blood serum was obtained by centrifugation at 3,000 rpm for 15 min, then stored in the freezer until performed. The blood serum was collected for the analysis of metabolites, including glucose, triglycerides, and blood urea nitrogen (BUN), which were estimated using diagnostic kits (glucose liquicolor, triglycerides liquicolor mono, urea liquicolor, respectively; Human) and a colorimetric method performed with a spectrophotometer.

#### Calculated economic aspects

Economic aspects in the dairy business can be determined through economic efficiency, feed efficiency, feed cost per liter of milk (FCLM), and income over feed cost (IOFC). Economy aspects were calculated using the formula:


$$\begin{array}{l} \text{Economic efficiency}=\frac{\text{Milk price }(\text{Rp})\times \text{Milk production }(\text{L})}{\text{Ration cost }(\text{Rp})}\\ \text{Feed efficiency}=\frac{\text{Milk production }(\text{L})}{\text{Dry matter intake }(\text{kg})}\\ \text{Feed cost per liter of milk }(\text{Rp}/\text{L})=\frac{\text{Ration cost }(\text{Rp})}{\text{Milk production }(\text{L})}\\ \text{IOFC }(\text{Rp})=\text{Selling price of milk }(\text{Rp})-\text{Ration cost per kg }(\text{Rp}) \end{array}$$

### Statistical analysis

#### Experiment 1

Data from 20 experimental units were analyzed with the PROC MIXED procedure using SAS On Demand for Academics (SAS Institute, Inc., Cary, NC, USA). The statistical model comprised five treatment diets as fixed effects and four different sources of rumen inoculum as random effects, defined as follows:


$${\text{Y}}_{\text{ij}}=\mu +\tau_{i}+\alpha_{j}+{ɛ}_{ij}$$

where Y*_ij_* represented the dependent variable, *μ* was the overall mean, *τ_i_* was the fixed effect of treatment diets (*i* = 1–5), *α_j_* was the random effect of rumen inoculum *j* (*j* = 1–4), and *ɛ_ij_* was the random residual error. Statistical differences were declared at p<0.05 and followed by the Duncan Multiple Range Test to compare means.

#### Experiment 2

All data were analyzed using the PROC MIXED procedure in SAS On Demand for Academics (SAS Institute, Inc.) according to the following model:


$${\text{Y}}_{\text{ij}}=\mu +\tau_{i}+\alpha_{j}+{ɛ}_{ij}$$

where Y*_ij_* represented the dependent variable, *μ* was the overall mean, *τ_i_* was the fixed effect of treatment diets (*i* = 1–4), *α_j_* was the effect of block *j* (*j* = 1–4), and *ɛ_ij_* was the random residual error. Differences were considered significant at p<0.05, and means were compared using the Duncan Multiple Range Test.

## RESULTS

### Experiment 1

#### In vitro fermentation characteristics and digestibility

The rumen pH, NH_3_ concentration, and population of protozoa, DMD and OMD are shown in Table 3. *In vitro* fermentation characteristics and digestibility were not affected by treatment diets (p>0.05). The DMD obtained in this study ranged from 58.65% to 65.18%, while the OMD ranged from 61.73% to 67.92%.

### Experiment 2

#### Environmental climate conditions

**Figure 1:**
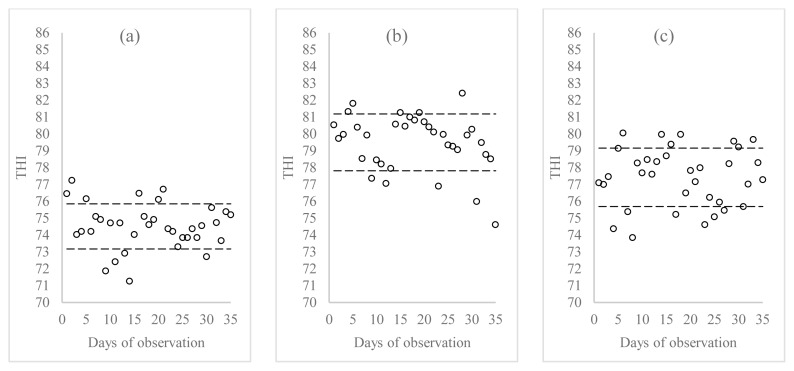
Temperature-humidity index (THI) on study farm: (a) morning, (b) afternoon, (c) evening.

Based on daily observations, the minimum temperature recorded in the morning was 22.2°C, while the maximum during the day reached 31.7°C. The average RH was higher in the morning (94.25%) compared to the afternoon and evening (72.47% and 82.33%, respectively). The THI values at the study farm are depicted in Figure 1. The calculated THI values ranged from 71 to 82, with an average THI of 77.15±2.59.

#### Feed intake

The nutrient intake, including DM, OM, CP, EE, CF, NFE, and TDN, is presented in Table 4. The results showed no significant difference between the treatment for DM intake of Sapera goats. This study’s average DM intake was 1,364.82 to 1,681.25 g/head/d, equivalent to 3.23% of BW. The DM intake based on metabolic body weight (BW^0.75^) ranged from 71.43 g/kg BW^0.75^ to 90.37 g/kg BW^0.75^. The treatment diet significantly affected the intake of CP, EE, CF, and NFE, while the intake of OM and TDN was unaffected by the treatment diet. Treatment R0 (basal diet) had the lowest intake of CP. Intake of CF and EE in R2 and R3 was lower than in treatment R0 and R1.

#### Milk production and milk quality

Milk production and milk quality are shown in Table 5. A significant impact on milk production was observed due to the treatment diets. Specifically, treatments R2 and R3 yielded elevated milk production levels (1.04 and 1.07 L/head/d) compared to treatments R0 and R1. Milk density was not affected by the treatment diets. Milk urea concentration was significantly different between treatment diets. Treatment R3 had the highest milk urea (22.25 mg/dL), while R0 (basal diet) obtained the lowest milk urea concentration (13.88 mg/dL). The treatment diets did not affect milk quality (%), including fat, solid non-fat (SNF), lactose, and protein. The milk yield component of SNF, lactose, and protein was higher in R2 and R3 compared to the R1 and R0 treatments.

The findings revealed notable variations in the C12:0 and C18:1 trans fatty acid levels among the treatment diets, whereas no distinctions were observed in other fatty acid profiles (Table 6). The milk fatty acid C12:0 and C18:2 *trans* decreased in treatment R1, R2, and R3 compared to the control (R0). The proportion of saturated fatty acids (SFA), unsaturated fatty acids (UFA), monounsaturated fatty acids (MUFA), and polyunsaturated fatty acids (PUFA) did not show significant differences between treatment rations. No significant differences were observed among the treatment diets regarding AI, HH, DI indices, and the PUFA to SFA ratio (Table 7).

#### Blood hematology and metabolite

The results showed that the treatment diets did not significantly affect the blood hematology of Sapera goats, including RBC, WBC, hemoglobin, and hematocrit (Table 8), and the WBC components (Table 9). The variance analysis showed significant differences among the treatment diets on glucose levels (p<0.05; Table 10). The R2 treatment resulted in highest blood glucose levels than the other treatments. Sulfur supplementation in R3 produced lower blood glucose levels. Meanwhile, triglycerides were not affected by the treatment diets. The BUN levels tend to increase in RDP-based rations either without or with the addition of cassava-NFC and sulfur (R1, R2, and R3) compared to basal diets (p<0.05).

#### Economy aspect

Economic aspects in this study can be measured through economic efficiency, feed efficiency, FCLM, and IOFC, presented in Table 11. Treatments R1, R2, and R3 increased feed costs per kg compared to the basal diet (R0). The reformulation diet (R1, R2, and R3) significantly affected feed efficiency and IOFC but did not significantly affect economic efficiency and FCLM. Treatment R3 showed the highest feed efficiency compared to other treatments. The selling price of goat’s milk received by Harokah Barokah Farm is Rp 40,000/L. The results showed that the R2 and R3 treatments resulted in the same and higher IOFC than the R0 and R1 treatments.

## DISCUSSION

### Experiment 1

#### In vitro fermentation characteristics and digestibility

Rumen pH is crucial for regulating microbial growth and the production of VFA and NH_3_. In this study, rumen pH values ranged from 6.58 to 6.68, within the normal range of 6.0 to 7.0 [17]. Diets based on RDP-protected (heated soybean), cassava-NFC, and sulfur inclusion did not significantly affect rumen pH, indicating a normal fermentation process.

The study revealed that NH_3_ concentrations ranged from 4.30 to 5.13 mM. The optimal range for NH_3_ concentration, was between 85 and 300 mg/L, equivalent to 4.9 to 17.6 mM [17]. Thus, using cassava-NFC, autoclaved soybean, and sulfur in the diets was deemed reliable for MP production and did not disrupt protein metabolism in the rumen. Gao and Oba [18] found decreased NH_3_ concentration in high NFC diets due to increasing OM fermented. High RDP diets raised NH_3_ levels. Heat treatment using an autoclave lowered NH_3_ concentration by creating resistant bonds that lower protein digestibility. The NH_3_ levels in this study were consistent with those of Rosmalia et al [6], who found no impact from sulfur supplementation. Factors influencing rumen NH_3_ include protein intake, feed degradability, retention time, and rumen pH.

In this study, the protozoa population ranged from 6.27 to 6.51 log cells/mL, higher than the normal range (5 to 6 log cells/mL) [17]. Protozoa are crucial for starch degradation in the rumen, and using cassava as a source of NFC increases their population. This aligns with a previous study that reported protozoa populations of 6.48 to 6.62 log cells/mL in cassava-NFC diets [7]. A consistent NFC level of 30% resulted in similar protozoa populations across different treatments. The treatment diets did not significantly impact the protozoa population, indicating that reformulating diets with RDP-protected (autoclaved soybeans), cassava-NFC, and sulfur did not eliminate protozoa. However, these results differ from Rosmalia et al [6], who found that RDP levels and sulfur supplementation reduced the protozoa population, with 0.1% and 0.2% sulfur supplementation lowering protozoa numbers. Easily degradable proteins and NFC levels can promote protozoa growth as they consume starch. Heat treatment, such as autoclaving, reduced feed degradability, inhibiting the rumen microbe’s growth, including protozoa.

DMD and OMD values in this study were comparable to the findings reported by Rosmalia et al [2] for a dairy cow ration (RDP 60% and NFC 30%), which exhibited digestibility levels of 65.21% (DMD) and 63.59% (OMD). The undifferentiated treatment diets on DMD and OMD suggested that the diet based on RDP, NFC, and sulfur supplementation did not compromise the digestion process of both DM and OM, affirming their safety for use. These results also align with a previous study conducted by Wulandari et al [19], which concluded that protein protected through heating at 120°C for 10 min did not impact digestibility. In addition, Chalupa [20] also reported that protected protein using an autoclave did not influence *in vitro* digestibility. Although a previous study reported that autoclave heat treatment might decrease DMD and OMD due to the Maillard reaction, which reduces rumen degradability and cellulolytic and proteolytic rumen microbial enzymes [3], this study did not observe such declines. The inclusion of NFC sources, such as cassava, can enhance rumen microbial degradability, compensating for the decrease in protein degradability. A high energy content in the diet was correlated with high DMD and OMD values. However, the outcomes of this study diverge Rosmalia et al [6], which reported that sulfur supplementation at levels of 0.1% to 0.2% enhanced the DMD and OMD values due to increasing rumen microbial population.

### Experiment 2

#### Environmental climate condition

Dairy goats regulate their body temperature despite environmental variations, which can affect feed intake and productivity. Maintaining optimal housing conditions is essential for their comfort. The thermal neutral zone for goats typically falls between 24°C to 30°C, with heat stress exceeding 32°C [21]. In this study, goats in the enclosure were within this zone, indicating no heat stress. The optimal RH for dairy goats is between 60% to 80%. The higher RH observed in this study was associated with its location on the slopes of Mount Salak, at an altitude of 587 masl. THI is an index used to assess the level of heat stress in dairy cattle by considering the temperature and RH of the air within the housing environment. Dairy goats are within the comfort zone at THI values below 72, while THI values between 72 and 79 indicate mild stress. The THI value observed in this study indicates mild to moderate stress conditions. Saanen crossbreds demonstrate greater resilience to moderate stress conditions than pure Saanen goats [22]. The THI value in Bogor Regency, with an altitude of 600 masl, was 77 [23].

#### Feed intake

The treatment diets that had an insignificant effect on DM intake showed that protected RDP-based rations without or with NFC and sulfur supplementation can be well received and consumed by livestock. A previous study also reported no difference in DM intake in dairy cows fed heat-protected soybeans [24]. Substituting rice bran for cassava as an energy source in dairy cow diets did not significantly affect DM and OM intake [25]. The level of feed intake is influenced by several factors, including the livestock condition (physiological status, pregnancy status, genetics, body condition score), feed quality (nutrient composition, particle size, and anti-nutrition), and environmental conditions (housing).

The lowest CP intake in R0 treatment showed that improving the quality of rations through reformulation based on protected RDP without or with cassava-NFC and sulfur supplementation positively impacted increasing nutrient intake. The increase in CP intake was also affected by the increased CP supply in the ration. The low CF and EE intake in R2 and R3 treatments was related to the DM intake and the fiber and fat content in the diet. Kanjanapruthipong et al [26] revealed that the NDF intake of dairy cows fed a total mix ration (TMR) with cassava was lower than cows with TMR corn, but the feed efficiency (4% fat-corrected milk per DM intake) was increased.

#### Milk production and milk quality

The synchronization of cassava-NFC with protected-RDP and sulfur supplementation in R3 treatment effectively produced a good performance from Sapera dairy goats. Protected-RDP-based rations plus cassava-NFC without sulfur supplementation also resulted in high milk production. Dias Júnior et al [24] reported that cows’ milk production increased due to feeding with protected soybeans. Protein protection increases the supply of amino acids in the small intestine so that nutrients for milk synthesis in the mammary glands are sufficient. The availability of NFC from cassava that rapidly degraded in the rumen increased the utilization of NH_3_ for MPS production, then increased milk production. Factors that affect milk production in dairy goats include livestock genetics, pregnancy status, number of kids per birth, udder size and shape, lactation period, and health status.

This study showed that milk density was not affected by the treatment. The milk density of the Saanen crossbreed ranged from 1.023 to 1.028 g/mL [27]. Several factors affect milk density, including lactation period, microclimate, feeding patterns, housing, season, and genetics. Milk urea concentration studies aimed at monitoring and controlling nutritional status, indicate protein metabolism and estimate nitrogen excretion in dairy livestock. In this study, R3 had the highest milk urea concentration (22.25 mg/dL), while R0 had the lowest (13.88 mg/dL). A low milk urea concentration indicates a deficiency in amino acid absorption, while a high urea concentration expresses an inefficient utilization of nitrogen. However, milk urea concentration in this study still did not reach the optimum value for high nitrogen use efficiency (28 to 32 mg/dL) [28]. The milk urea concentration in goat’s milk varied from 11.90 to 67.50 mg/dL. Milk urea concentration also positively correlated with BUN. A previous study reported that feeding low RDP to dairy cows reduced the component of milk urea and BUN [16]. Urea concentration in milk was influenced by genetics, lactation period, pregnancy status, season, BW, litter size, milk production, and milk nutrient composition.

The results of the milk nutrient component (milk fat and protein) in this study showed a premium category for fresh goat milk per the Thai Agricultural Standard No. 6006 [29]. This study showed that R2 and R3 treatment produced high milk SNF, lactose, and protein. The high production of milk nutrient components, also in line with high milk production, contributed to R2 and R3 treatments. SNF in milk is influenced by lactose and protein. Increasing the energy level increased SNF and milk protein levels as a component of SNF. This is in line with the improvement in feed quality in R1, R2, and R3, which has an impact on increasing the production of SNF, lactose, and milk protein components. Increasing fiber intake enhanced the SNF and fat milk content.

Milk fatty acids synthesis in ruminants follows 2 pathways. Firstly, the mammary glands engage in de novo synthesis of C≤15 and a portion of C16 through the fermentation of polysaccharides, resulting in the production of acetate or β-hydroxybutyrate. Secondly, a portion of C16 and long-chain fatty acids (C≥17) are synthesized from adipose tissue reserves and dietary lipids. In comparison to cow and sheep milk, goat milk exhibited a higher abundance of C6:0, C8:0, C10:0 fatty acids, conjugated linoleic acid and a lower n-6 to n-3 ratio [30]. This study’s reformulation diet decreased C12:0 and C18:1 trans fatty acids. A previous study found that goats fed extruded soybeans reduced their C12:0 fatty acid profile [31]. This decrease is due to an increased supply of long-chain fatty acids in a protected RDP-based diet by inhibiting *de novo* synthesis of medium-chain fatty acids such as C12:0 in the mammary glands. The protected RDP (soybean) diet rich in PUFA added by cassava-NFC rich in pectin reduced C18:1 trans through increased biohydrogenation so that it did not indicate a decrease in milk fat. Chilliard et al [32] described that goat’s milk was not sensitive to changes in the rumen biohydrogenation pathway, which caused C18:1 trans-10 to replace C18:1 trans-11 as the primary intermediate compared to cow’s milk.

An RDP-based diet with heat-protected soybean and NFC synchronization from cassava and sulfur supplementation did not change the proportions of SFA, UFA, MUFA, and PUFA. Mohd Nor et al [27] reported that the proportion of SFA and UFA in Saanen goat-fed concentrate with 17.13% of CP was 69.28% and 30.61%, respectively. Another study revealed that goats’ SFA, MUFA, and PUFA components range from 59.0% to 74.0%, 19.0% to 36.0%, and 2.6% to 5.6% [33]. The SFA component in this study was low, but the PUFA component was high. In contrast, previous research showed that the proportion of SFA decreased while MUFA and PUFA increased in goats fed 10% up to 20% extruded soybeans in the ration [31]. Hence, protecting feed protein by heating prevented the biohydrogenation process from occurring in the rumen, increasing PUFA production. Fatty acids in milk are influenced by animal individuality, genetics, milk production, lactation period, biosynthetic and biohydrogenation process in the rumen, feed composition, forage-to-concentrate ratio, fat supplementation, and rearing management.

The health index of milk fatty acids (AI, HH, and DI) in this study was not affected by the treatment diet. Chen and Liu [34] reported that the AI indices for the Saanen goats ranged from 2.77±0.08, while for the Nguni and Boer, 2.34 and 2.60, respectively. The AI index is associated with specific fatty acids (C12:0, C14:0, and C16:0) known for their atherogenic properties. The AI value in this study was lower than in the previous study due to the high production of the PUFA component (19.82% to 22.36%). The high UFA component can be attributed to a low AI value due to the high utilization of forage in the diet [15]. Hypocholesterolemic fatty acids are components associated with lowering blood serum cholesterol levels. The HH values observed in this study remained within the normal range, as defined by Bonanno et al [35] (1.26 to 2.09).

The DI ratio serves as an indicator of Δ^9-desaturase activity in the mammary glands. The DI ratio in this study was lower (0.14 to 0.61) than Ferrand-Calmels et al [36] reported, where the DI ratio in goat’s milk ranges from 0.5 to 6.67. The DI ratio found in this study was low, which can increase the SFA content of milk. Nonetheless, the SFA content of milk in this study was still relatively normal and tended to be lower than previously reported [27]. PUFA-rich diet altered and increased the expression of the Δ^9^-desaturase gene, which also impacts the DI ratio. A PUFA to SFA ratio above 0.45 was needed to prevent human coronary heart disease and cancer. Although not significantly different, the PUFA/SFA ratio in R2 and R3 treatments was relatively high (0.44 and 0.45). Variations in health index value are influenced by differences in feed, forage-to-concentrate ratio, and fat supplementation.

#### Blood hematology and metabolite

This study found that the treatment diet did not affect the blood hematology parameters (RBC, WBC, hemoglobin, hematocrit, and leukocyte differentiation) in Sapera goats. The RBC, WBC, hemoglobin, hematocrit, and leukocyte differentiation were in the normal range, according to Weiss and Wardrop [37]. This suggests that the diet based on RDP, regardless of the inclusion of cassava-NFC and sulfur supplementation, did not interfere with the blood hematology of Sapera goats. Furthermore, the hematology of Sapera goats that received the treatment diet showed that their blood parameters remained within the normal range, indicating that the goats were healthy [37].

Blood metabolites describe the nutritional status of dairy goats associated with performance and health status. Blood glucose indicates energy status in ruminants. Blood glucose levels in the R2 treatment were the highest among the treatments, supported by a sufficiently high supply of carbohydrates by using cassava in R2 treatment. The addition of sulfur to R3 led to decreased blood glucose levels. A previous study reported that supplementation of inorganic sulfur MgSO_4_ decreased blood glucose levels in dairy cows [38]. Sulfur supplementation alters the metabolic process of hydrogen (H_2_) from CH_4_ to hydrogen sulfide (H_2_S), which contributes to the regulation of blood glucose levels in the body’s homeostasis. Blood glucose and triglyceride levels are positively correlated with milk fat content. However, in this study, the high blood glucose level observed in the R2 treatment did not exhibit a significant difference in the increase of milk fat content. Blood glucose serves as a source for milk lactose synthesis. Blood glucose levels in this study were still within the normal range (52.03 to 59.29 mg/dL) [39]. Sapera goats typically exhibit blood glucose levels ranging from 44 to 78 mg/dL.

Blood triglyceride levels are associated with lipid metabolism in the livestock body. In this study, the treatment diets did not affect blood triglyceride levels and remained within the normal range, according to Bagnicka et al [40]. Manuelian et al [41] reported that Saanen goats kept in the Mediterranean area had blood triglyceride levels ranging from 15.40 to 56.20 mg/dL. In dairy cattle, blood triglyceride levels were twice as high during the dry phase compared to the lactation phase due to the lipolysis of adipose tissue caused by energy deficit and hormonal changes before parturition. A previous study stated that increasing the supply of RUP, particularly with sufficient methionine availability, positively impacted on very low density lipoprotein synthesis, leading to increased blood triglyceride levels in young goats [42].

BUN levels in this study ranged from 19.65 to 35.13 mg/dL and were still within the normal range, according to Kohn et al [43]. The high BUN level in R1 was attributed to using protected soybeans without adequate supplementation of carbohydrate sources. It can be seen in Table 10 that a diet based on RDP, when balanced with the inclusion of cassava-NFC and sulfur supplementation (R2 and R3), resulted in lower BUN levels compared to R1. High BUN levels are closely related to increased NH_3_ concentrations in the rumen and are associated with increased milk urea. The lower BUN level observed in R2 corresponded to a lower average milk urea concentration (18.50 mg/dL) compared to R1, although this discrepancy did not reach statistical significance. The inclusion of cassava as an energy source in the R2 treatment can enhance the utilization of nitrogen by rumen microbes, reduce the gluconeogenic use of amino acids, and decrease the release of excess nitrogen into the environment. The R3 treatment resulted in the highest BUN and milk urea levels. These levels can be attributed to the positive correlation between high milk production and increased BUN and milk urea nitrogen due to high-producing livestock with a greater nutrient demand. A high intake of RUP and amino acids could increase BUN levels. Factors that affect BUN levels are feed, genetics, and physiological status.

#### Economy aspect

The economic efficiency found in this study did not differ among the treatments. Economic efficiency is determined based on factors such as milk production, milk prices, animal feed consumption, and ration costs. The highest feed efficiency found in this study was the R3 treatment. This indicates that the protected RDP, cassava-NFC, and sulfur-based rations can be consumed, digested, and metabolized effectively, increasing milk production and feed efficiency. Factors influencing feed efficiency in dairy goats include the fat milk correction factor, BW, DM digestibility, fiber content in the feed, and proportion of forage [44]. The IOFC in R2 and R3 treatments was higher than in R0 and R1 treatments. This indicates that providing protected-RDP-based diets with NFC, either with or without sulfur supplementation (R2 and R3), offered goats more significant economic benefits than other diets.

## CONCLUSION

In conclusion, incorporating cassava-NFC and autoclaved soybean in the ration preserved digestibility, despite not enhancing *in vitro* fermentability. Rations based on RDP, NFC, and sulfur led to higher milk production and improved nutrient efficiency without adverse effects on animal health. These findings emphasize the potential benefits of precision ration formulations considering RDP and NFC in enhancing dairy goat performance and the overall economic aspects of dairy farming. We recommend that dairy goat farmers, under conditions similar to those in our research station, supplement their basal diet with 12% autoclaved soybeans, 9% cassava NFC, and 0.11% sulfur.

## Figures and Tables

**Table 1:** Experiment 1: Experimental diet for *in vitro* analysis

Parameters	Treatment^1)^				

	P0	P1	P2	P3	P4
Feed ingredients (% DM)					
Elephant grass	50.00	50.00	50.00	50.00	50.00
Cassava meal	0.00	7.50	7.50	7.50	7.50
Rice bran	6.00	2.50	2.75	2.75	2.50
Pollard	9.33	4.25	3.33	3.33	3.15
Molasses	6.00	4.00	4.00	4.00	4.00
Copra meal	8.23	9.25	9.25	9.25	9.25
Palm kernel meal	5.70	7.75	9.35	9.35	9.35
Soybean	0.00	0.00	5.00	0.00	0.00
Autoclaved soybean	0.00	0.00	0.00	5.00	5.00
Corn gluten meal	2.25	2.05	2.00	2.00	2.00
Corn gluten feed	6.13	6.30	3.85	3.85	3.85
DDGS	5.28	5.30	1.88	1.88	1.88
Dicalcium phosphate	0.10	0.10	0.10	0.10	0.10
CaCO_3_	1.00	1.00	1.00	1.00	1.00
Na_2_SO_4_	0.00	0.00	0.00	0.00	0.43
Nutrient contents					
Dry matter (%)	90.96	91.52	92.09	91.82	91.40
Ash (% DM)	12.15	11.95	11.49	11.92	12.00
Crude protein (% DM)	13.79	13.63	13.67	13.52	13.43
RDP (% DM)	8.56	8.41	8.50	8.41	8.34
RUP (% DM)	5.23	5.22	5.17	5.11	5.09
Ether extract (% DM)	2.60	1.74	1.75	3.02	3.01
Crude fiber (% DM)	19.99	19.53	18.53	20.43	18.32
NFE (% DM)	51.46	53.15	54.57	51.11	53.25
TDN (% DM)	59.24	59.05	60.56	59.24	61.46

DM, dry matter; DDGS, distiller’s dried grains with soluble; RDP, rumen degradable protein; RUP, rumen undegradable protein; NFE, nitrogen-free extract; TDN, total digestible nutrient; NFC, non-fiber carbohydrate.

1) P0, control diet (without cassava-NFC and autoclaved soybean); P1, P0+7.5% cassava-NFC; P2, P0+7.5% cassava-NFC and 5% soybean; P3, P0+7.5% cassava-NFC and 5% autoclaved soybean; P4, P0+7.5% cassava-NFC, 5% autoclaved soybean, and 0.1% sulfur.

**Table 2:** Experiment 2: Experimental diet for Sapera dairy goat

Parameters	Treatment^1)^			

	R0	R1	R2	R3
Feed ingredients (% DM)				
Dwarf elephant grass	40.00	40.00	40.00	40.00
Tofu dregs	21.00	21.00	21.00	21.00
Commercial concentrate	38.04	26.04	14.64	14.04
Cassava meal	0.00	0.00	9.00	9.00
Protected soybean	0.00	12.00	12.00	12.00
Corn gluten meal	0.00	0.00	2.40	2.52
Premix	0.24	0.24	0.24	0.24
Dicalcium phosphate	0.12	0.12	0.12	0.12
CaCO_3_	0.60	0.60	0.60	0.60
Na_2_SO_4_	0.00	0.00	0.00	0.48
Nutrient contents				
Dry matter (%)	37.97	39.20	41.27	41.33
Ash (% DM)	11.32	10.47	8.94	8.96
Crude protein (% DM)	13.76	17.50	16.71	16.69
Ether extract (% DM)	3.65	5.64	3.85	4.44
Crude fiber (% DM)	20.34	19.82	16.91	16.77
NFE (% DM)	50.93	46.58	53.60	53.13
TDN (% DM)	60.84	65.85	68.90	69.63

DM, dry matter; NFE, nitrogen-free extract; TDN, total digestible nutrient; NFC, non-fiber carbohydrate.

1) R0, basal diet (farm diet); R1, R0+12% autoclaved soybean; R2, R0+12% autoclaved soybean and 9% cassava-NFC; R3, R0+12% autoclaved soybean, 9% cassava-NFC, and 0.11% sulfur.

**Table 3:** Experiment 1: *In vitro* fermentation characteristics and digestibility

Parameters	Treatment^1)^					SEM	p-value

	P0	P1	P2	P3	P4		
Rumen pH	6.65	6.67	6.63	6.58	6.66	0.049	0.203
NH_3_ (mM)	4.30	4.33	5.13	4.41	4.33	0.211	0.701
Protozoa (log cell/mL)	6.28	6.27	6.51	6.34	6.37	0.052	0.573
DMD (%)	59.79	63.64	65.18	58.65	63.18	0.975	0.124
OMD (%)	62.98	66.90	67.93	61.73	65.62	0.951	0.115

SEM, standard error of the means; DMD, dry matter digestibility; OMD, organic matter digestibility; NFC, non-fiber carbohydrate.

1) P0, control diet (without cassava-NFC and autoclaved soybean); P1, P0+7.5% cassava-NFC; P2, P0+7.5% cassava-NFC and 5% soybean; P3, P0+7.5% cassava-NFC and 5% autoclaved soybean; P4, P0+7.5% cassava-NFC, 5% autoclaved soybean, and 0.1% sulfur.

**Table 4:** Experiment 2: The nutrient intake of Sapera dairy goat fed the experimental diet

Parameters (g/head/d)	Treatment^1)^				SEM	p-value

	R0	R1	R2	R3		
Dry matter	1,364.82	1,590.11	1,681.25	1,577.97	69.173	0.427
Organic matter	1,325.62	1,460.03	1,573.45	1,469.03	62.187	0.439
Crude protein	231.17^b^	340.34^a^	345.74^a^	315.95^a^	16.300	0.049
Ether extract	59.03^b^	104.14^a^	76.33^b^	77.69^b^	5.638	0.011
Crude fiber	247.24^ab^	272.57^a^	227.58^b^	224.03^b^	11.766	0.007
Nitrogen-free extract	704.88^c^	742.98^bc^	923.80^a^	851.35^ab^	39.915	0.028
Total digestible nutrient	920.81	1,169.16	1,297.31	1,201.75	57.313	0.117

SEM, standard error of the means; NFC, non-fiber carbohydrate.

1) R0, basal diet (farm diet); R1, R0+12% autoclaved soybean; R2, R0+12% autoclaved soybean and 9% cassava-NFC; R3, R0+12% autoclaved soybean, 9% cassava-NFC, and 0.11% sulfur.

a–c Mean values within a row with different superscript letter differ significantly (p<0.05).

**Table 5:** Experiment 2: Milk production and milk quality of Sapera dairy goats fed the experimental diet

Parameters	Treatment^1)^				SEM	p-value

	R0	R1	R2	R3		
Milk production (L/head/d)	0.60^c^	0.83^b^	1.04^a^	1.07^a^	0.070	0.002
Milk density (g/mL)	1.030	1.027	1.028	1.027	0.472	0.316
Milk urea (mg/dL)	13.88^c^	19.25^b^	18.50^b^	22.25^a^	0.879	0.000
Milk composition (%)						
Fat	6.04	7.89	6.38	7.25	0.38	0.312
Solid non fat	8.81	8.45	8.49	8.52	0.08	0.843
Lactose	3.96	3.82	3.83	3.84	0.04	0.900
Protein	4.17	4.00	4.02	4.03	0.04	0.840
Milk component yield (g)						
Fat	37.94	65.80	68.55	77.40	5.216	0.194
Solid non fat	54.42^c^	72.11^b^	91.13^a^	93.45^a^	6.008	0.003
Lactose	24.45^c^	32.58^b^	41.15^a^	42.18^a^	2.715	0.003
Protein	25.74^c^	34.11^b^	43.17^a^	44.26^a^	2.848	0.003

SEM, standard error of the means; NFC, non-fiber carbohydrate.

1) R0, basal diet (farm diet); R1, R0+12% autoclaved soybean; R2, R0+12% autoclaved soybean and 9% cassava-NFC; R3, R0+12% autoclaved soybean, 9% cassava-NFC, and 0.11% sulfur.

a–c Mean values within a row with different superscript letter differ significantly (p<0.05).

**Table 6:** Experiment 2: Milk fatty acid profiles of Sapera dairy goats fed the experimental diet

Parameters (% milk fat)	Treatment^1)^				SEM	p-value

	R0	R1	R2	R3		
C4:0	1.24	1.35	0.51	1.52	0.326	0.774
C6:0	0.43	0.27	0.28	1.72	0.359	0.453
C8:0	0.32	0.18	0.28	0.80	0.130	0.384
C10:0	8.26	10.42	9.27	10.21	0.538	0.572
C11:0	0.25	0.19	0.23	0.34	0.030	0.336
C12:0	4.82^a^	3.56^b^	3.02^b^	3.81^b^	0.220	0.017
C13:0	0.18	0.11	0.13	0.19	0.017	0.246
C14:0	3.89	3.26	3.14	3.36	0.160	0.480
C14:1	0.54	1.63	0.97	0.60	0.327	0.679
C15:0	0.48	0.48	0.47	0.72	0.055	0.413
C15:1	0.12	0.08	0.17	0.27	0.050	0.614
C16:0	19.74	19.65	19.11	17.67	0.651	0.711
C16:1	0.69	0.54	0.76	0.67	0.066	0.703
C17:0	0.23	0.18	0.19	0.21	0.013	0.617
C17:1	0.20	0.14	0.39	0.37	0.081	0.704
C18:0	10.35	13.58	11.78	10.11	0.638	0.146
C18:1 *trans*	7.68^a^	3.62^b^	4.09^b^	2.87^b^	0.616	0.010
C18:1 *cis*	19.00	21.23	20.41	18.67	0.766	0.696
C18:2 *trans*	1.10	0.84	0.73	0.83	0.053	0.100
C18:2 *cis*	14.21	12.62	16.31	13.40	0.880	0.413
C20:0	0.23	0.17	0.48	0.31	0.076	0.573
C18:3n6	0.38	0.21	0.31	0.34	0.046	0.723
C20:1	0.14	0.07	0.14	0.38	0.063	0.364
C18:3n3	1.85	1.49	2.02	1.94	0.105	0.211
C21:0	0.75	0.79	0.87	0.97	0.061	0.284
C20:2	0.65	0.51	0.46	1.08	0.108	0.166
C22:0	0.16	0.17	0.31	0.28	0.058	0.796
C20:3n6	0.28	0.37	0.25	0.71	0.110	0.434
C22:1n9	0.09	0.06	0.12	0.26	0.049	0.512
C20:3n3	0.25	0.13	0.18	0.63	0.116	0.436
C20:4n6	0.45	0.57	0.58	0.89	0.085	0.288
C23:0	0.09	0.04	0.07	0.19	0.027	0.271
C22:2n6	0.16	0.15	0.18	1.17	0.251	0.434
C24:0	0.10	0.06	0.07	0.18	0.026	0.361
C20:5n3 (EPA)	0.15	0.09	0.35	0.38	0.086	0.647
C24:1	0.23	0.45	0.40	0.90	0.103	0.114
C22:6 (DHA)	0.34	0.74	1.00	1.04	0.187	0.568
∑SFA	51.59	54.52	50.32	52.86	1.060	0.487
∑UFA	48.41	45.48	49.68	47.14	1.060	0.487
∑MUFA	28.59	27.76	27.32	24.74	0.761	0.438
∑PUFA	19.82	17.72	22.36	22.40	1.031	0.236

SEM, standard error of the means; EPA, eicosapentaenoic acid; DHA, docosahexaenoic acid; SFA, saturated fatty acids; UFA, unsaturated fatty acids; MUFA, monounsaturated fatty acids; PUFA, polyunsaturated fatty acids; NFC, non-fiber carbohydrate.

1) R0, basal diet (farm diet); R1, R0+12% autoclaved soybean; R2, R0+12% autoclaved soybean and 9% cassava-NFC; R3, R0+12% autoclaved soybean, 9% cassava-NFC, and 0.11% sulfur.

a,b Mean values within a row with different superscript letter differ significantly (p<0.05).

**Table 7:** Experiment 2: Health index of milk fatty acids of Sapera dairy goats fed the experimental diet

Parameters	Treatment^1)^				SEM	p-value

	R0	R1	R2	R3		
AI	0.83	0.80	0.71	0.77	0.040	0.750
HH	1.37	1.48	1.73	1.74	0.085	0.285
DI	0.14	0.61	0.34	0.21	0.130	0.638
PUFA/SFA	0.39	0.33	0.45	0.44	0.027	0.238

SEM, standard error of the means; AI, atherogenicity index; HH, hypocholesterolemic/hypercholesterolemic; DI, desaturase index; PUFA/SFA, ratio of polyunsaturated fatty acids and saturated fatty acids; NFC, non-fiber carbohydrate.

1) R0, basal diet (farm diet); R1, R0+12% autoclaved soybean; R2, R0+12% autoclaved soybean and 9% cassava-NFC; R3, R0+12% autoclaved soybean, 9% cassava-NFC, and 0.11% sulfur.

**Table 8:** Experiment 2: Hematology of Sapera dairy goats fed the experimental diet

Parameters	Treatment^1)^				Standard^2)^	SEM	p-value

	R0	R1	R2	R3			
RBC (×10^6^/mm^3^)	12.54	12.44	10.18	10.20	8–18	6.603	0.267
WBC (×10^3^/mm^3^)	9.02	9.01	8.84	9.88	4–9	1.086	0.974
Hemoglobin (g %)	9.93	9.15	9.65	9.30	8–12	0.300	0.846
Hematocrit (%)	26.75	28.00	29.25	27.75	22–28	0.844	0.653

SEM, standard error of the means; RBC, red blood cells; WBC, white blood cells; NFC, non-fiber carbohydrate.

1) R0, basal diet (farm diet); R1, R0+12% autoclaved soybean; R2, R0+12% autoclaved soybean and 9% cassava-NFC; R3, R0+12% autoclaved soybean, 9% cassava-NFC, and 0.11% sulfur.

2) Data from Weiss and Wardrop [37] .

**Table 9:** Experiment 2: White blood cell component of Sapera dairy goats fed the experimental diet

Parameters (%)	Treatment^1)^				Standard^2)^	SEM	p-value

	R0	R1	R2	R3			
Lymphocytes	50.20	52.31	50.46	53.52	50–70	0.754	0.477
Neutrophils	38.40	36.96	35.93	36.62	30–48	0.764	0.769
Eosinophils	7.31	7.43	8.43	5.78	1–8	0.613	0.488
Monocytes	2.95	1.98	3.51	2.14	0–4	0.326	0.422
Basophils	1.15	1.32	1.68	1.94	0–1	0.179	0.513

SEM, standard error of the means; NFC, non-fiber carbohydrate.

1) R0, basal diet (farm diet); R1, R0+12% autoclaved soybean; R2, R0+12% autoclaved soybean and 9% cassava-NFC; R3, R0+12% autoclaved soybean, 9% cassava-NFC, and 0.11% sulfur.

2) Data from Weiss and Wardrop [37].

**Table 10:** Experiment 2: Blood metabolites of Sapera dairy goats fed the experimental diet

Parameters (mg/dL)	Treatment^1)^				Standard	SEM	p-value

	R0	R1	R2	R3			
Glucose	52.28^b^	54.22^ab^	59.29^a^	52.03^b^	50–75^2)^	1.200	0.046
Triglyceride	19.50	15.25	15.06	21.04	8–27^3)^	1.688	0.637
Blood urea nitrogen	19.65^b^	35.13^a^	27.41^ab^	33.81^a^	25–38^4)^	2.145	0.033

SEM, standard error of the means; NFC, non-fiber carbohydrate.

1) R0, basal diet (farm diet); R1, R0+12% autoclaved soybean; R2, R0+12% autoclaved soybean and 9% cassava-NFC; R3, R0+12% autoclaved soybean, 9% cassava-NFC, and 0.11% sulfur.

2) Data from Kaneko et al [39].

3) Data from Bagnicka et al [40].

4) Data from Kohn et al [43].

a,b Mean values within a row with different superscript letter differ significantly (p<0.05).

**Table 11:** Experiment 2: Economy aspect and feed efficiency of Sapera dairy goats fed experimental diet

Parameters	Treatment^1)^				SEM	p-value

	R0	R1	R2	R3		
Feed cost (Rp/kg)	2,096	2,552	2,803	2,839	-	-
Economy efficiency	8.52	8.22	8.75	9.59	0.436	0.328
Feed efficiency	0.45^c^	0.52^bc^	0.61^ab^	0.68^a^	0.033	0.007
Feed cost per litre of milk (Rp/L)	5,263	4,989	4,658	4,208	267	0.328
Income over feed cost (Rp)	21,199^c^	29,169^b^	36,931^a^	38,395^a^	2,604	0.002

SEM, standard error of the means; NFC, non-fiber carbohydrate.

1) R0, basal diet (farm diet); R1, R0+12% autoclaved soybean; R2, R0+12% autoclaved soybean and 9% cassava-NFC; R3, R0+12% autoclaved soybean, 9% cassava-NFC, and 0.11% sulfur.

a–c Mean values within a row with different superscript letter differ significantly (p<0.05).
